# Mentalization-Based Treatment From the Patients’ Perspective – What Ingredients Do They Emphasize?

**DOI:** 10.3389/fpsyg.2019.01327

**Published:** 2019-06-12

**Authors:** Katharina Teresa Enehaug Morken, Per-Einar Binder, Nina Margot Arefjord, Sigmund Wiggen Karterud

**Affiliations:** ^1^Bergen Clinics Foundation, Bergen, Norway; ^2^Department of Clinical Psychology, Faculty of Psychology, University of Bergen, Bergen, Norway; ^3^Solli District Psychiatric Centre (DPS), Bergen, Norway; ^4^Norwegian Institute for Mentalizing (IM), Bergen, Norway

**Keywords:** substance use/addiction, qualitative interviews, hermeneutical-phenomenological, personality disorder, thematic analyses, mentalization-based treatment

## Abstract

**Objective:** The aim of this study was to explore how patients with personality disorder (PD) and substance use disorder (SUD) experience mentalization-based treatment (MBT), in particular what they consider useful and less useful elements of the therapy.

**Method:** Semi-structured qualitative interviews with 13 participants were conducted. Participants were interviewed on their experience of the different elements of MBT, their experience of working in the transference, and their view on MBT as a whole. Thematic analyses were performed within a hermeneutical-phenomenological epistemology, with an emphasis on researcher reflexivity.

**Results:** The following themes were found in the material: “I am not alone,” “Taking blinders off,” “Just say it,” “The paradox of trust,” and “Follow me closely.” Three of these themes concerned therapist interventions; these involved addressing the relationship with the patients, addressing negative or unspoken feelings in the sessions, and validating and tolerating patients’ affect. Two themes concerned group therapy experiences; these were the experience of sameness with co-patients in group and the experience of discovering different perspectives in group.

**Conclusions:** Patients’ experiences of useful elements in MBT resonate with theoretical tenets of (borderline) personality pathology, in particular attachment disturbances and emotional dysregulation. Patients highlight what we would label working in the therapeutic relationship, addressing transferential and counter-transferential processes explicitly, emotional validation, and enhancing mentalizing in its own right.

## Introduction

Mentalization-based treatment (MBT) with patients who suffer from both personality disorder (PD) and substance use disorder (SUD) is an area where we still lack sufficient empirical evidence. So far, only two clinical trials on MBT with PD\SUD patients have been published (Morken et al., 2017b; Philips et al., 2018). In our pilot trial with 18 female PD\SUD patients, we investigated both feasibility and experiences of the treatment (Morken et al., 2017a,b). In the explorative qualitative study, 13 female patients were interviewed on their experiences of change, with a special reference to mental states and feelings (Morken et al., 2017a). From the perspective of the patients, the changes were pervasive. In this study with the same patients, we focus on how they experienced the actual MBT treatment. We already know they experienced meaningful change, and now we investigate what elements of MBT they highlight for their change.

PD\SUD patients are known for being challenging in treatment. These challenges can be summarized within three areas: relationally, health risks and attendance. Relationally these patients pose challenges for therapists because of their difficulties with mentalizing and relational functioning. The alliance is often more negative (Olesek et al., 2016). Therapists, who lack a strong therapeutic frame, are drawn into counter-transferential processes that increase the risk for iatrogenic damages (Fonagy and Bateman, 2007). Therapists can both reject patients and provoke alliance ruptures, or take over their mentalizing so that they decrease the agency of their patients. Furthermore, there are substantial health risks for these patients. Borderline patients are often suicidal, in one longitudinal study 10% of the cohort ended up completing suicide (Paris and Zweig-Frank, 2001). Among PD\SUD patients, the substance use is often more hazardous, and suicidal tendencies are also increased (Links et al., 1995; Paris and Zweig-Frank, 2001). SUD patients, especially those involved with harder drugs (Nyhlen et al., 2011), are at risk for early death. These two diagnoses combined pose an obvious danger for their health. Unfortunately, these dual diagnoses are also at a higher risk for not attending psychotherapy. They will more often drop out of therapy, and if they do stay in treatment they will more often have negative outcomes (Thomas et al., 1999; Bornovalova and Daughters, 2006; Tull and Gratz, 2012). Thus, to summarize, PD\SUD combined pose severe challenges in psychotherapy and the need for knowledge on how therapy works for these patients is important.

From psychotherapy research, we have some knowledge on mechanisms of change within psychotherapy. Therapists seem to account for much of the change themselves, as there are variations between different therapists within the same treatment models. Some of the hallmarks of these therapists are the ability to form strong alliances, facilitating interpersonal skills, professional self-doubt, and engagement in practicing therapy skills outside of therapy (Wampold, 2015). From systematic reviews on psychotherapy, findings are that alliance rupture repairs have a moderate positive effect on outcome in psychotherapy (Eubanks et al., 2018) and that alliance in treatment of borderline PD is related to positive outcome (Barnicot et al., 2012). Both poor affective communication and poor alliance are related to dropout from therapy for borderline PD patients (Barnicot et al., 2011). In a qualitative meta-analysis of 109 studies on patient experiences of therapy, five clustered themes were found: (1) change is a holistic process that involves multiple faculties; (2) a caring and understanding therapist is essential; (3) structure in therapy maneuvered flexibly; (4) explicitly discussing client-therapist role differences and engage in active collaboration; and (5) clients agency and self-healing processes (Levitt et al., 2016.). More specifically in research on MBT, one study compared MBT group therapy with psychodynamic group therapy and found that MBT groups had a much higher frequency of interventions that require a mentalizing response (Kalleklev and Karterud, 2018). Another study demonstrated that interventions in accordance with the MBT manual would increase mentalizing in the patients’ following response (Möller et al., 2016). A third study demonstrated a link between mentalizing and symptomatic improvement (De Meulemeester et al., 2017). One study found that clear communication, accurately perceiving the process, being MBT adherent, practicing a not-knowing stance and an affect focus were all therapist skills that promoted completion of therapy (Philips et al., 2017).

**Figure 1:**
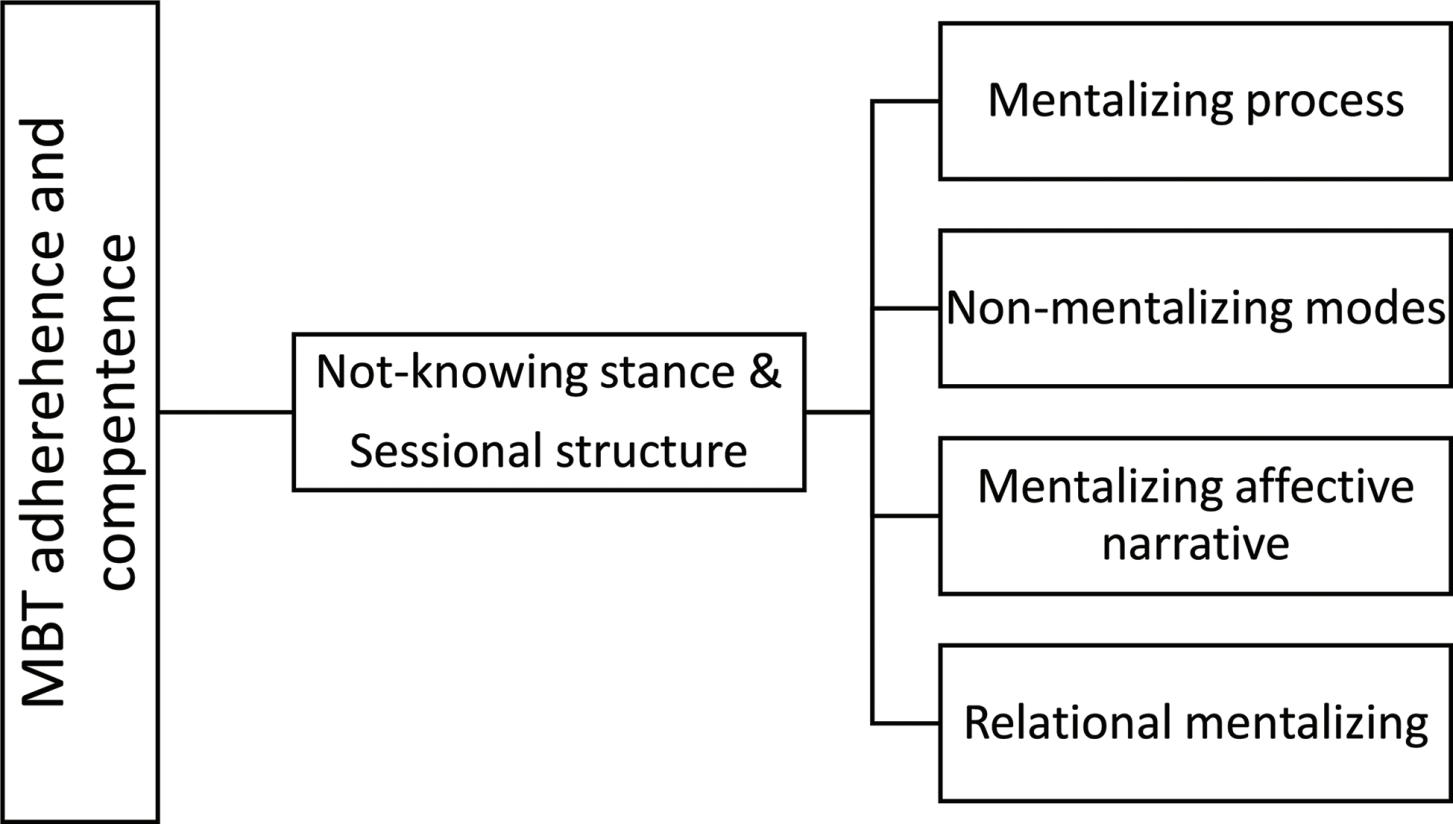
MBT adherence and competence scale.

MBT is a conjoint therapy with weekly group and individual therapy. In MBT, the proposed mechanism of change is an “exclusive focus on the BPD patients current mental state while activating the attachment relationship” (Fonagy et al., 2015). Attention to the relationship between therapists and patient is crucial. Furthermore, a specific focus on mentalizing is key. Bateman has summarized the main interventions in MBT (see Figure 1) as comprised of two general domains and four major component domains (Bateman, 2018). The general domains are underlying quality assets of sessions and are “sessional structure” and “the not-knowing stance.” The four major component domains, which are specific therapist interventions, are: “mentalizing process,” “non-mentalizing modes,” “mentalizing affective narrative,” and “relational mentalizing.”

To explain, “the not knowing stance” is a stance where clinicians are aware that mental states in self and other are opaque and they do not jump to conclusions about their patients statements. “Sessional structure” means following a trajectory from what was agreed upon; focus on mentalizing, having an explicit conversation about what theme should be addressed, and closing down this theme within the time of the session. “Mentalizing process” entails focusing on the process of mentalizing. “Non-mentalizing modes” is to intervene adequately when patients are in non-mentalizing modes. “Mentalizing affective narrative,” means that clinicians should focus on the narratives with an emphasis on affect. “Relational mentalizing,” means to have a focus on the relations between therapist and patient and between patients in the group.

**Figure 2:**
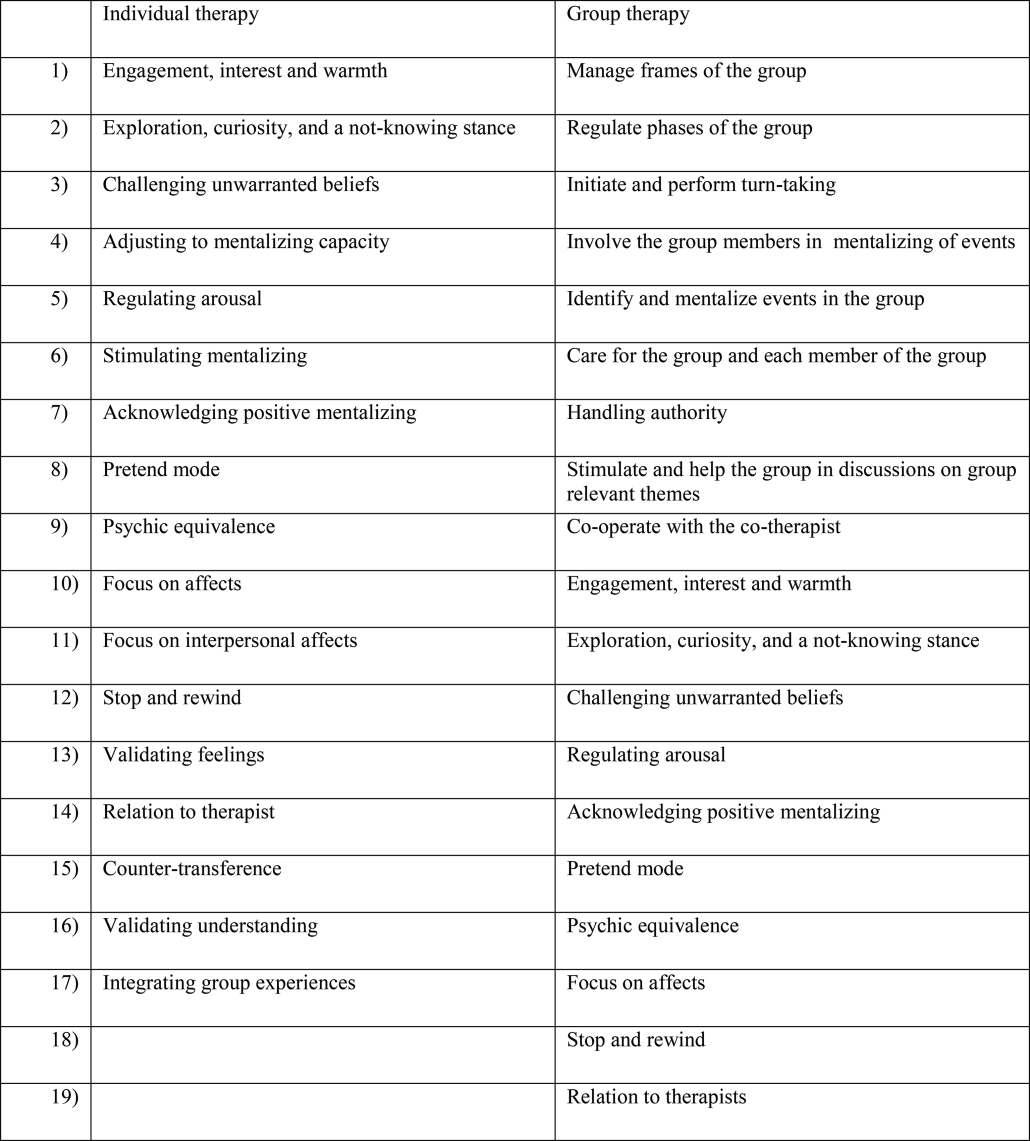
Mentalization-based therapy adherence and quality scale (MBT-AQS).

In Norway, three manuals for all parts of MBT (psychoeducational, group, individual) have been published (Karterud and Bateman, 2010; Karterud, 2011, 2012), including a mentalization-based therapy adherence and quality scale (MBT-AQS). Reliability research for the MBT-AQS (including an adaptation for MBT group therapy) has been performed inside and outside of its original research context (Karterud et al., 2013; Folmo et al., 2017; Simonsen et al., 2018). The MBT-AQS is composed of two items as shown in Figure 2.

In addition to the manualized descriptions of high quality MBT, we have some empirical findings pointing to aspects of MBT that could be important. A qualitative study of MBT group therapy found that good quality group therapist strategies are to be able to maintain a balance between an authoritative and a not knowing stance (Inderhaug and Karterud, 2015). Other studies on groups (Kalleklev and Karterud, 2018; Karterud, 2018) have underlined the significance of challenging unwarranted assumptions and posing so-called “demand questions” (e.g., why do you believe that your mother said that?). A qualitative interview study on patients’ experiences with MBT found that patients wanted direction and structure (Dyson and Brown, 2016). A qualitative investigation of patient experiences found that they experienced groups as challenging and underlined the importance of building trust (O Lonargain et al., 2017). A qualitative study that involved timeline analyses of three participants (a lived experience study) found that being understood broke their cycle of self-hatred and social exclusion (Johnson et al., 2016). Recently, a qualitative investigation of high vs. low quality MBT concluded that hallmarks of high-quality MBT in which high-rated therapists investigated maladaptive patterns were more challenging and brought patients out of their comfort zone (again, challenging and “demanding”). This therapy style facilitated the alliance and created a constructive therapeutic process (Folmo et al., 2019). From our investigation of patients’ experiences of change, we found that they experienced meaningful changes in their ability to cope with mental states in self and gained a more flexible understanding of others (Morken et al., 2017a). The question that remains is how they experienced that MBT assisted them in this change process. The present study expands on our first study and investigates in depth how patients from a successful MBT trial experience the treatment and which elements of the treatment they highlight as meaningful.

## Aims

Patients who have been through therapy can shed light on processes of which therapists themselves are not aware. With patients that are sensitive to therapeutic ruptures and vulnerable for dropping out of therapy, there is a good rational for exploring their subjective perspectives. Ideally, we can tailor our therapeutic approaches so that we reduce drop out and hinder iatrogenic damage. How do patients with PD/SUD experience MBT? What elements of MBT do they describe as useful?

## Materials and Methods

### Methodological Approach

We aimed to find patients’ experience of the therapeutic processes by utilizing an open and flexible strategy in the collection and analyses of data. We performed qualitative interviews with open-ended questions with a special focus on the following: individual therapy, group therapy, the relationship with therapists, and MBT as a whole. Three of the authors (1–3) conducted the interviews, first author did not interview any own patients. Three authors were involved clinically in the pilot trial, one as a therapist (1) and two as supervisors (3, 4). Thematic analyses within a hermeneutic-phenomenological epistemology were performed (Laverty, 2003; Binder et al., 2012). Our own theoretical ideas on psychotherapy gave ground for the reflexive dialogue with the material. We chose a hermeneutical-phenomenological approach as we were both interested in patients’ experiences and how our perspectives would influence the analyses. In thematic analyses, these processes are made overt and transparent, so that other researchers can follow the process easier and so that we acknowledge that context and interpretation influence the findings (Finlay, 2012). The findings are embedded within a specific context and within a specific mind-set of us, the authors. To disentangle these mind sets with an eye on what these patients are trying to convey will give an idea on what patients can experience as participants in MBT.

### Treatment

The Bergen Clinic Foundation decided in 2009 to do a pilot project to investigate whether MBT was a promising method in the treatment of patients with dual diagnosis PD\SUD. Eighteen female patients were included in the pilot and received up to 3 years of MBT. The treatment followed the manual as suggested from the original authors with two exceptions. First, all patients in this pilot had access to an MBT informed social counselor who could help with social and economic needs; second, the therapy lasted up till 36 months (instead of 18 months). All therapists were specialized in MBT with introductory (three days) and advanced courses (8 days over 1 year) of MBT. MBT adherence was not measured. Therapists had weekly video supervision with a supervisor expert in MBT and monthly video supervision with an external supervisor expert in MBT. All therapists in the pilot were clinical psychologists, except for one group therapist who was a specialized nurse in the treatment of addiction. The social counselor also participated in the weekly and monthly supervision.

### Participants

Thirteen patients were recruited to the interview study approximately 2 years after the end of treatment. All patients were female and had comorbid personality disorder (PD) with substance use disorder (SUD). All were raised in Norway and had similar cultural background. Mean age was 28 (SD 6.52), range of age was 20–41, and mean level of education after junior high school was 2.5 years (SD 1.6). At start of treatment, the patients had the following diagnostic characteristics. The distribution of SUD’s was as follows: harmful use\dependency of alcohol (*n* = 7), harmful use\dependency of cannabinoids (*n* = 3), harmful use\dependency of amphetamine (*n* = 3), harmful use\dependency of benzodiazepines (*n* = 5), and harmful use\dependency of opiates (*n* = 4). Six patients had more than one SUD at start of treatment. All patients had maladaptive traits within the category of BPD. The distribution of PDs was as follows: borderline personality disorder (*n* = 10), antisocial personality disorder (*n* = 2), avoidant personality disorder (*n* = 3), dependent personality disorder (*n* = 1), schizotypal personality disorder (*n* = 1), paranoid personality disorder (*n* = 2), obsessive-compulsive personality disorder (*n* = 1), histrionic personality disorder (*n* = 1), and PD NOS (*n* = 2). Seven patients had more than one PD (range 2–3 PDs). No patients had the full profile of schizoid personality disorder or narcissistic personality disorder.

### Researchers

One author has a PhD and is a clinical psychologist with a specialization in addiction (1), one is a clinical psychologist with a specialization in addiction (3), one is a professor in clinical psychology (2), and one is a professor in psychiatry (4). Three authors are specialized in MBT (1, 3, and 4), and one author is specialized in qualitative research and hermeneutical-phenomenological approaches (2). Three authors were involved in the pilot project as individual therapists (1) and supervisors (3, 4). The first author was individual therapist of six participants prior to the study; the first author interviewed none of these participants.

### Recruiting Participants

In the process of recruiting participants to the pilot project, we went out broadly in the Bergen Clinic Foundation (outpatient, inpatient detoxification, and inpatient long-term units) and asked for female patients that were considered difficult to treat with the tentative diagnosis of BPD. Eighteen patients entered the pilot project. At the time of follow-up, approximately 1 year after treatment, all 18 patients were contacted by letter and telephone and invited to participate in a follow-up assessment. Thirteen patients agreed to participate. For their participation, they received a gift certificate of 500 NOK (approximately 50 Euros\60 US Dollars).

### Data Collections Method

Three interviewers (1, 2, and 3 author) conducted 13 interviews. Semi-structured, exploratory, and in-depth interviews were performed in order to investigate patients’ experiences with MBT. Interviews had a mean duration of 64 min and were transcribed in their entirety.

### Data Analysis

The analyses were conducted following the procedure of thematic analysis within a hermeneutical-phenomenological framework (Binder et al., 2012). The process of analyses involves going back and forth between the different phases of the analyses described below:

1. Three interviewers noted their immediate impressions after the interviews. The goal of this was to establish a basic sense of the heterogeneity and homogeneity of participants’ experiences and to increase reflexive awareness.
2. All researchers read all of the transcribed material to obtain a basic sense of the participants’ experiences. This phase also involved a gradual recognition of personal and professional preconceptions.
3. Examining those parts of the text relevant to the research question, the first author identified separable categories and used NVivo software to organize the material (Bazeley and Jackson, 2013).
4. The first and second authors then rearranged the categories into broader themes based on the data’s implicitly expressed meaning.
5. All authors turned back to the overall text to check whether voices and points of view should be added to the themes. Reorganization of some themes and consensual discussions took place in this phase of analyses.
6. All authors agreed upon the themes that thus made the material for the findings reported.

### Reflexivity

Reflexivity is defined as “a process of continually reflecting upon our interpretations of both our experience and the phenomenon being studied so as to move beyond the partially of our previous understandings” (Finlay and Gough, 2003, p. 108) and is a key ingredient in qualitative research. When the data material is comprised of subjective experiences and is in its nature textual, there is a need for an analytic strategy that enhances trustworthiness and transparency. In qualitative methodology, reflexivity is that tool. Reflexivity should be utilized within three areas, these are own bias and assumptions, theoretical bias and assumptions, and the ideological landscape that psychotherapy exists in (Binder et al., 2016). The main complication with performing research within own clinical context, and the therapy you are enthusiastic about, is of course a positive bias. These issues were in the back of our heads during analysis, and when something in the data seemed to be confirming “the excellence of MBT,” it would go an extra round before being categorized as a finding. It was also very beneficial for the analysis that one author (2) was not in the field of MBT and could then have a more open mind about the interpretation of patients experiences.

There are other pitfalls to be aware of, for instance, first author has a personal preference for direct, transparent, and outspoken therapists. This was also one of the findings, in the study, that patients prefer that type of therapist. Thus, there is a risk for contamination here that we believe was avoided because of the multiple authors cooperating in the analysis (Binder et al., 2016).

Furthermore, to get in contact with the experience of the participants, time and patience were the key. Reading and rereading, defining and categorizing, going back to the text in a listening mode, and repeating the whole cycle again. We believe that it is important, especially when the researchers are close to the data, that the analysis is given sufficient time and focus, so that the process of bracketing own assumptions and the art of listening to the voices of the participants get sufficient time and focus.

### Ethics

The Regional Committee for Medical and Health Research Ethics (Region West) situated at the medical faculty of the University of Bergen approved the study. All participants received written and oral information about the purpose of the study and their voluntary participation. Written informed consent was obtained from the participants prior to the interview. They were informed that they could withdraw from the interview at any time.

## Results

Five themes regarding patients’ experiences of MBT were found in this material. All participants’ names are fictitious.

### Themes

#### Taking Blinders Off

This theme deals with participants’ discovery of the existence of other perspectives than their own, and in addition, the surprising experience was that this broader mindset could actually help with calming them down in situations where their emotions were high. The group therapy is especially highlighted as a context that could broaden their perspective. They describe the importance of listening to co-patients and therapist’ view on a situation and then experiencing the surprising discovery of a different mindset than their own. At first, these different points of view were confusing, but after a while, several participants experienced that in their own mind this ability of juggling with different perspectives appeared automatically and had a calming effect. Suzannah found other patient’s view on her own life situations very helpful; she also found the transition to a broader view calming. Her issues with men were often a topic:

I could sit in the group crying, sometimes hysterical, and then everyone, I told them about my crisis, and then everyone gave their opinion about that situation, and often from a different perspective that I could not see, I had these blinders on, and it gave me hope to listen to them, that maybe he meant that by doing this, instead of me thinking that he was cynical, manipulative and mean and wanted to hurt me, that they turned it around and viewed it from another angle, this has helped me immensely, and with time it made me able to think like that myself, to be able to calm myself down.

For the participants, the notion that other people think differently and have other frames of reference was surprising, and this gave them hope. Diana was surprised when she discovered that others think differently from her during the psychoeducational group at the beginning of therapy.

So I joined this group and we got these assignments, you know, if a woman and a man is watching a football game together and then the woman just took off when they had this date, right, and then she got drunk, and for me it was like of course, she is jealous, and I was certain it was like that, and then the others, no it was like this and this, and then a third, and then suddenly, god, there are so many reasons, and then I suddenly got others point of view on the same problem, and I was not used to that, I was only thinking in one direction you know.

This change from tunnel vision to a broader view expanded their view on their own life history, on people they dislike, and on their own responsibility in conflicted situations. Camilla used the group to help her understand the mind of a coworker in her new job and then took on responsibility to change the conflict.

There was an incident when I started working, and there was this girl, really pissy towards me and very nice and happy with everyone else, and then I went to the group and I told them, you know there is this girl, I don’t know what to do, and then I don’t know if it was the therapist who said, imagine that she is having a bad day, or that she deals with strangers poorly, that she doesn’t know how to react with new people, and I brought that with me, went back to work and was just super nice to her, it took ten minutes and then she was like that with me.

The ability to experience other minds as different from their own paved way for accepting and respecting others and themselves in a new way. This transition from my view to your view and the acceptance of others as someone with different perspectives was not an easy process, and it involved some pain. For Susannah, it was hard to realize that her point of view was not necessarily the only valid view in a conflict between her and a co-patient in group.

I remember I was so provoked because she spent all her time on talking about her dog, and I just couldn’t understand how an animal could be important, seriously you are talking about your dog and I have lost my whole family and almost my life, and you are talking about taking walks in parks, why are you talking about this stuff, but you know, again I had to swallow it and listen, and I had to realize that for this person it was important, and I had to accept and respect that.

In summary, this theme is about the discovery of a world of minds where previously one perspective was the rule and also experiencing that this ability to think in multiple perspectives at the same time has a calming effect when under emotional distress.

#### I Am Not Alone

This theme is about identifying with other women in the group and discovering that you are darkest and ugliest secrets are not that ugly after all. The women seemed to bond with each other and feel like a community of equals, and this provided a safe base for them while exploring difficult aspects of themselves in therapy. By identifying with other women with similar problems, patients achieved a sense of self-worth where they before suffered with shame and a sense of being bad. Maria gained a new perspective on the validity of own thinking by listening to other patients input in the group.

I have been very bad in giving input, it is because I have been insecure if it is … I have thought one thing and then wanted to say it, but then thought no its completely wrong, it sounds stupid, and then someone else has said what I wanted to say, and then it just sounded very good or logical, but when I thought it inside me I couldn’t say it you know, but I got confirmation that it wasn’t completely off what I was thinking.

By listening to other women reveal their difficulties, patients discovered and were surprised that other women had the same difficulties as themselves. By this discovery, the women had to restructure their own self-image because the other person is someone they judge to be an okay human being, thus their own dark thoughts and feelings about themselves no longer made sense. The problems they are identifying with are not primarily addiction or substance use-related issues, which also these women have in common, but more the emotional pain and suffering that they endured in their lives. It is a process where they moved from a position of being alone in the world with an identity that is abnormal and extreme, to a position where they are not alone and not that bad. Julia felt normalized after listening to co-patients.

I remember I got help by listening to others in the group, things that I thought was completely abnormal was normal after all, it was little things, like one who woke up in the middle of the night wanting to use drugs, and then she felt she was the only one in the world because it was so quiet, and later she figured out that this was anxiety, I noticed that episode, because I thought about many episodes where I didn’t understand my own reactions and then I figured out that it could be anxiety.

This entailed deeper acceptance for own faults, less guilt for past history, and a more positive view on themselves, their thoughts and feelings. For Diana, having similar themes as other group members, helped her to forgive herself for childhood traumas.

I thought it was only me, but then we address different stuff, like, I have a father that will not have anything to do with me, and suddenly another in the group also had a father, and what to do with him you know, should she care or not, and there I am, should I care or not, and it’s probably my fault, you know, oh god all the fault that you carry, you think that everything is your fault, and then suddenly, hello, you were a child, it was not your fault, you couldn’t do anything.

They experienced that viewing other women from within did something positive to their own view on themselves from outside. Group therapists probably recognize this as collective pseudo-mentalizing or what we could call the “tea-party phenomenon” because it could often happen in groups when patients are joining in on each other in saying that they feel exactly like the others do.

In summary, this theme is about how patients achieved more self-worth and self-acceptance by bonding and identifying with women with similar problems. This opened up an experience of participating in a common humanity and prevented the feeling of being alienated and alone. The main experience here was that by viewing other women from within patients achieved new views on themselves from outside.

#### Just Say It!

This theme deals with participants’ experiences of thoughts and feelings that are for various reasons an obstacle to constructive therapeutic processes and in addition are difficult to utter. We could call this “elephant in the room” situations, because patients describe them as emotionally challenging, like themes that probably were present in all participants’ mind, or that it was very present in their own mind, but still were ignored by the ones responsible, the therapists. These dangerous mind states can occur in both the group and individual sessions. These unstated thoughts and feelings can reside both within the participants themselves, in their co-patients, and in their therapists. In general, the idea that comes through the interviews is that unuttered thoughts and feelings are something negative if not stated aloud and that not saying them aloud is a hindrance for therapy. They described the need to dig them out; utter them while they are fresh, state thoughts aloud so that they do not grow and become bigger and more negative, and process them in the here and now. Timing is important; it needs to happen in the here and now, in the moment so to speak of the incident. Natalie was upset with the group therapists when they failed to address an obvious conflict between two co-patients in one of the sessions; she worried for both parts of the conflict including the aggressor.

That they didn’t address it before next time; that they didn’t just deal with it then and there, it would have been better for the one that did it, if you wait until next time it will not be the same, you cannot change it in the same way I think.

The content of these unspoken thoughts and feelings was often difficult to say aloud, it implied some kind of risk to the relationship with the therapists or co-patients. Examples of relevant content are, for example, negative feelings about therapists or patients, important aspects of own persona that feels necessary to be honest about, quirks in the relationship with co-patients or therapists, or obvious violations to the contract of therapy as, for example, expressing very positive attitudes about drug use. Moreover, when therapists failed to address clear “elephants” in the room, participants were disappointed and at unease. Robyn was worried about a co-patient when the therapists did not intervene on “drug-talk.”

and they were very careful in the way they intervened, because I could see in some, especially a young girl who was very chaotic and on and off, I could see that she was very influenced by all this talk about drugs and alcohol, it was not good, that they did not strike down on it immediately, that can be devastating.

The patients needed that therapists took responsibility for repairing relational ruptures both between patients and between therapists and patients. When the therapists managed to address the “elephants in the room,” this was experienced as helpful. For Diana, repairing conflicts with other patients with the help of the group therapist felt good and it was a new experience for her.

You could be annoyed in the groups as well you know, but then you could just tell the therapist and address it next time, they didn’t trust each other, they didn’t know each other so well, right, and then some things that people said could sting the others, and that happened often, and then it was like, they just have to address it immediately, and that was really great, instead of saying I hate you, you know, or stare at each other, I just said I didn’t like what she said, and the therapist would just address it, Diana didn’t like what you said, and then we talked about it, instead of people being bitter at each other, that was fucking great.

They also suggested that having the possibility to talk things over with their individual therapist after group was necessary because of the tensions, conflicts, and themes that group experiences could evoke in them. Dealing with it when the material was still fresh was helpful.

In summary, this theme underlines the importance of creating a therapeutic space where all thoughts and feelings are okay to express. The reason for this is that negative feelings and thoughts will grow and become and obstacle to therapy if not adequately expressed. Negative feelings toward therapists and co-patients should be asked for and dealt with. The responsibility to make a wide and open therapeutic space lies with the therapists, and individual therapists can help patients to process difficult themes.

#### The Paradox of Trust

This theme contains the paradox of patients who at one hand disliked talking about the relationship with the therapist and at the same time felt safer with therapists who explicitly addressed the relationship between them. The patients described that talking about the relationship with their therapist was difficult, weird, or otherwise unpleasant. At the same time, patients felt safer with therapists that explicitly addressed the relationship. Diana felt very safe with her therapist, and at the same time, she disliked when they talked about whether she trusted him:

Diana: “Yes if I trusted him or if I felt that I could talk freely and all that.” Interviewer: “and how was that for you, to be asked about such things?” Diana: “that was shit; it was stupid, because he knew that I trusted him, so it was really unnecessary to ask.”

There are three important aspects of this theme. First necessity, some patients experienced that talking about the relationship with the therapist was a necessity for managing to endure the therapy. The intimacy of one-to-one therapy with the individual therapists might provoke many negative thoughts and feelings. Not all patients managed to link that talking about this makes them safer, but still they experienced safety only with the therapists who actually asked them about how they feel with them in the room. Natalie also had a therapist that asked her about how she felt in the relationship but in a similar way as Diana, Natalie did not have anything specific to address:

Interviewer: “So how was it with the individual therapist, did you talk about how you felt with her or were there sometimes that you didn’t feel safe with her? Natalie: “that’s not something that I can be bothered to sit and talk about, because with her, I haven’t felt unsafe or something like that.”

Second, not daring to address it by themselves, several patients had negative feelings about their therapists but saying that aloud was beyond their comfort-zone. Robyn did not remember that the relationship was addressed with her first MBT therapist, and at the same time, she found no room to talk about the issues between her and the therapist.

Robyn: “She was my age, and I found that a bit difficult, she seemed insecure at times, and I felt that I knew more about alcohol and treatment then her”. Interviewer: “Did you say any of this to her?” Robyn: “No, I didn’t have the guts to do that.”

In addition, if they had negative feelings about their therapists they often put the blame and responsibility for these feelings on themselves. Patients might have experienced extreme vulnerability when they found themselves in a relationship with a therapist. As demonstrated by Eva who even on filling out alliance forms felt vulnerable and often denied to answer the questions.

Eva: “I was not interested in trying to determine whether it was a therapist doing her job or if she actually cared.” Interviewer: “And this was something that you talked about with your therapist?” Eva: Yes and that was okay I guess, but I still do not see why I have to make a judgment on whether the therapist like me, that’s just too much stuff to think about. I liked the therapist and that is what matters. And I am so bad in guessing whether people like me or not. I assume that everyone dislike me. Interviewer: So you still don’t see the usefulness of that?” Eva: “No it makes me feel vulnerable.”

Third, the basic need for a therapeutic space where all thoughts and feelings about the relationship are welcome to exploration is demonstrated by this theme. Therapists must take responsibility for this space to be created because the patients did not feel comfortable doing that by themselves. Therapists who admit responsibility for ruptures in the relationship created safety for these patients. For Eva, it was something new that she was not to blame when there were conflicts in the relationship with the therapists.

What I noticed was that there was less conflict with the therapist than with earlier therapists, and that when there was a conflict some effort were made to try and fix it, earlier I have experienced that they meant that it was my responsibility to fix the problems without even considering that they could have been wrong at all.

In summary, this theme describes the paradox that these participants experienced when the relationship with the therapist is addressed, and it underlines the necessity of actually addressing the relationship often enough so that a space for exploring ruptures could be created.

#### Please Follow Me Closely

One final theme that came up in the material was that patients described important moments of validation from their therapists as vital for feeling safe in therapy. What seems to be the headline of this is that the therapists take their perspective and intervene according to whatever position the patient is in. It also contains the important notion of therapists who did not act out. Natalie talked about being understood and listened to by her therapist, instead of a therapist who was acting out:

Now with my therapist I can say how I feel, but the other one, I couldn’t say that I was not feeling good because then she would omit me immediately, so when I went there I could not say how I felt because I knew she would come with that stuff. But now my therapist is not like that, for example if there’s something, she would ask me if that’s something I would like to do, instead of having to do it.

We could describe this as a dichotomy between being professional and caring where the patients experience that the good therapists find a middle way between professional distance and familiar closeness. These therapists are experienced as therapists who knew when to keep distance but still managed to demonstrate that they care. Hannah experienced more safety when talking about emotional themes because her therapist kept some distance during exploration.

she realized early on that if I were to be able to talk about sensitive stuff, then we had to put a lid on it at the same time, she had to sort of look away, because if not I would not open up, so that instead of earlier psychologists who like really wanted to talk about it, why did you feel this way or that way, and did you start crying because you talked about it, that made it hard to talk about, here it was more like, do you want me to look away?

These therapists communicated accept for the patients’ different manners. They also endured strong emotions without becoming agitated or acting concretely. Suzanna explained that her therapist did exactly what she needed and by that, she felt understood and seen.

And I was just sitting there screaming, and he just like … what do you need me to do now, and I just said I need you to tell me that things will be alright, that things will be better, and then he did that, and I got calm 100%, and it was like, he is not freaking out, he is not scared, he does not make me feel completely insane.

They were matching their interventions so that patients felt seen, heard and tolerated. There was also an aspect of timing what they said and did, so that they were behaving in a foreseeable manner. The patients experienced that their therapists calmed them down when they were agitated. In addition, the patients felt sometimes very understood when the therapist actively told them what their thoughts about them were. These words coming from the therapist could even be integrated in their identities long after. The counterpart of this validating and transparent therapist described above was someone who mismatched the patient. These therapists intervened artificially or with an agenda. Robyn explains how the therapist could miss the essence of the story, for example, because of focusing too much on irrelevant details:

The two therapists were different, one was more thoughtful, she was very, she spent a lot of time on wondering, down to the last detail, I felt it could be a little too much focus on irrelevant details and weird questions, she was more interested in mentalizing then in the actual situation that we were discussing, I liked better the other therapist.

Therapists who negated the experience of the patient or who became as agitated as the patient could provoke alliance ruptures. Kara felt violated by the therapist’s focus on grasping different perspectives when there was a new patient joining the group therapy, and the theme was as serious as a history of violence.

someone came into group, and I had a history with her, real serious incident where they knocked the face in on my boyfriend at the time, I could not recognize him afterwards, and then she stood there laughing, hitting me and interrogating me, and then she enters the group, and I felt that it was like this is mentalizing, now you are going to mentalize that this will work out, I felt really uncomfortable.

In summary, this theme describe patients’ experiences of therapists who were matching their position in the moment, tolerated, and accepted their position in the moment and communicated clearly and directly so that they were predictable for the patients.

### Summary of Findings

In this study, we found five themes describing from the patients perspective, therapeutic processes in MBT. The themes are “I am not alone,” “Taking blinders off,” “Just say it,” “The paradox of trust,” and “Follow me closely.”

**Figure 3:**
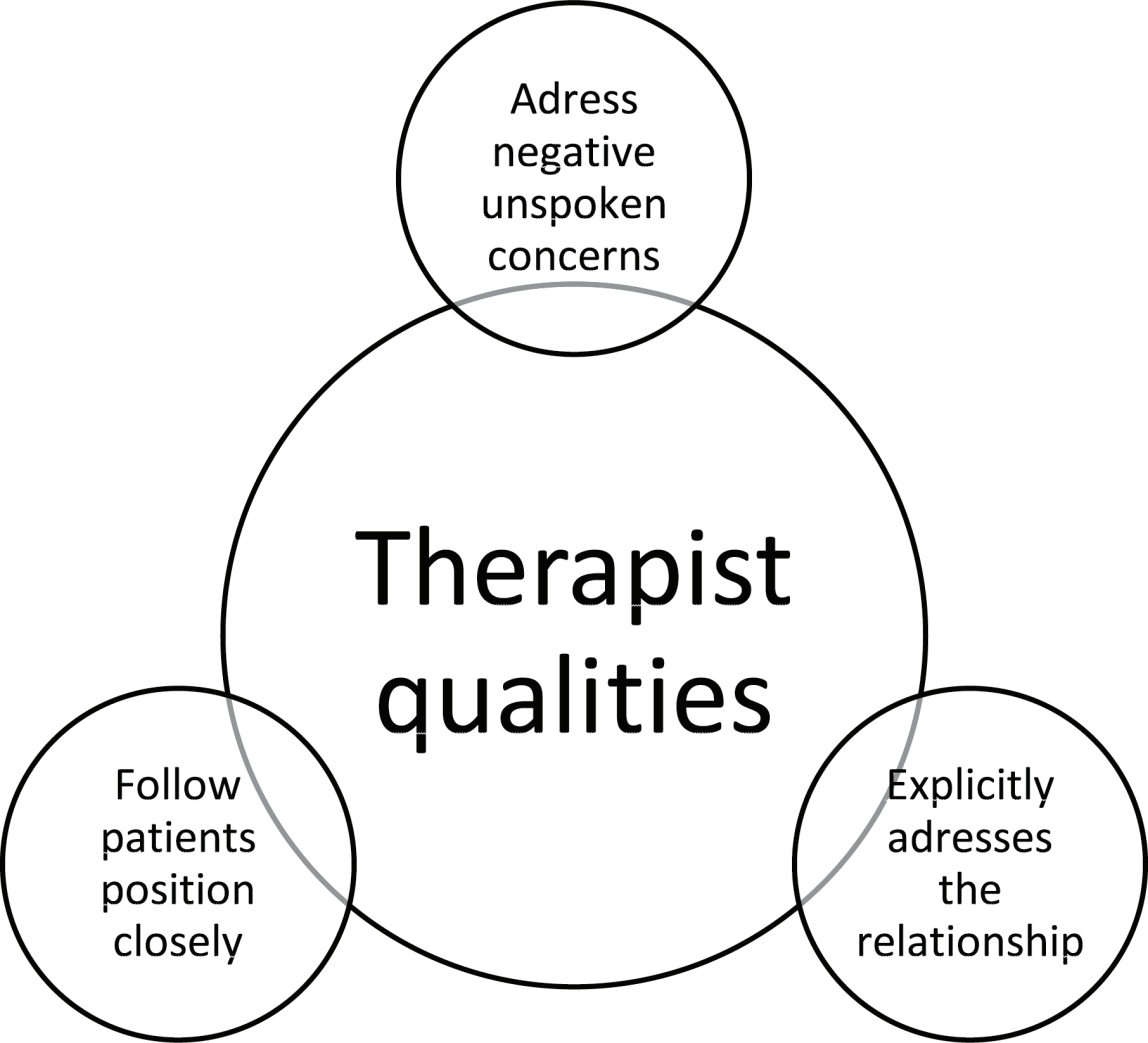
Therapist qualities according to patients’ point of view.

Figure 3 demonstrates therapist strategies that these patients experienced as vital to set in motion therapeutic processes. First, it was experienced as important that therapists followed closely their perspective here and now through explicit validation and by demonstrating that they could deal with affect. Second, in order to have a safe relationship with the therapists where there can be room for exploring, it appeared to be necessary that therapists explicitly addressed the relationship between them and the patients. Third, patients suffered when the therapists did not explicitly address clear “elephants in the room.” These elephants in the room were often conflicts, co-patients who uttered destructive thoughts, or other negatively charged themes that went on between individuals in the group sessions.

**Figure 4:**
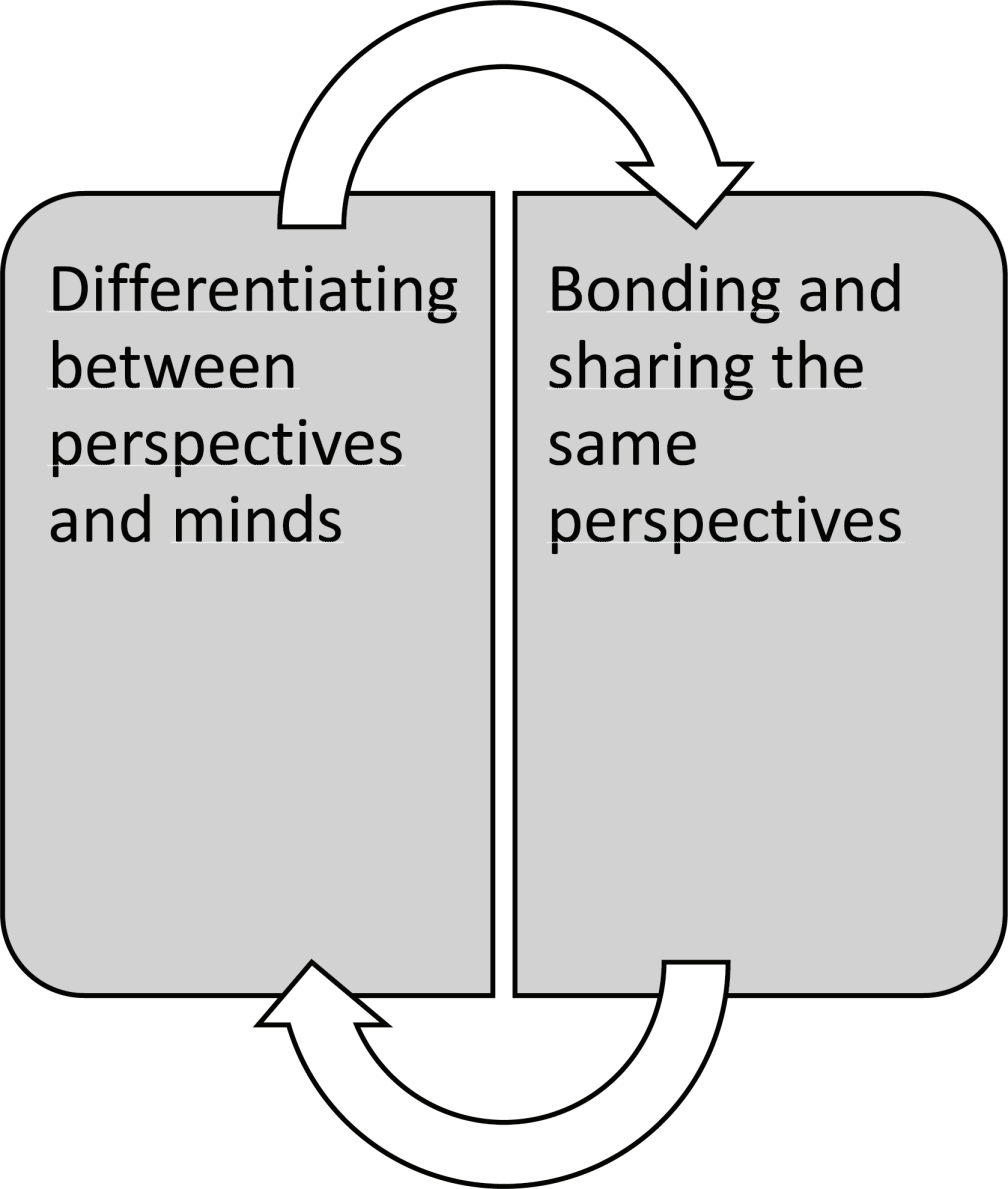
Group therapy processes that lead to meaningful change according to patients’ point of view.

Figure 4 demonstrates their point of view regarding therapeutic processes in group therapy alone. Their perspective implies that therapists should tailor group therapy sessions so that patients can switch back and forth between bonding and differentiating processes. The first was necessary for the experience of safety in groups, and identifying with others had potent effects on their negative self-images. However, in order to be able to get over the tunnel vision these patients experienced, identification was not enough. Therapists needed also to point out differences in minds and ensure that differentiating occurred. This juggling of perspectives had also a demonstrably effect on regulating affect in the moment.

## Discussion

The findings of this study expand on existing knowledge on patients struggling with attachment and affect regulation and poor social cognition. We found that from the patients’ point of view, therapist who tolerates strong emotions, who addresses negative unspoken concerns, and who addresses the relationship between themselves and their patients is the style perceived as most helpful. Furthermore, patients describe oscillation between two opposite group processes as useful in gaining normalcy and new mindsets. The group processes were bonding and sharing perspectives and the experience of having different perspectives. In summary, these findings coincide with the proposition that mentalizing is a mechanism of change in MBT. Furthermore, the findings resonate well with some of the proposed interventions in MBT, like the not-knowing stance, affect focus, and mentalizing the relationship (Bateman, 2018). From the MBT-AQS (Karterud et al., 2013), more interventions seem to coincide with the findings: engagement, interest, and warmth; validating feelings; regulating arousal; adjusting to mentalizing capacity. Affect regulation and interpersonal functioning are core elements of what patients with PD/SUD struggle with, hence our findings pose no major surprises. What we do notice though is that our patients put demands on therapists to address the elephant in the room or say aloud negative phenomena in the here and now. This therapeutic style is also well recognizable as recommended in MBT (Bateman, 2010–2018; Bateman and Fonagy, 2016). Should this clinical intervention perhaps also be explicitly mentioned in the adherence scales? Managing countertransference is a recommended intervention, but that does not capture the importance of addressing elephants in the room, even if it can imply to do so. For patients who struggle with mentalizing deficits and large trust issues with others, therapists who are demonstrably direct, have a transparent mind, say aloud the obvious, is probably calming and ensuring. From the patients’ point of view, this is an important quality of therapists that make them feel safe.

### Therapist Qualities

According to our findings, good therapists know when to keep distance and when to come close, they are explicit about the content of own mind, they address the elephant in the room, and they tolerate strong affect. They put focus explicitly on the relationship between themselves and their patients. These findings resonate well with existing knowledge on therapist factors where the ability to form strong alliances and facilitative interpersonal skills is found to be essential (Wampold, 2015). The importance of therapists, who form strong alliances, is vital for patients. Lack of affective communication and the alliance have been found to be of importance in the prevention of drop out in treatment for BPD patients (Barnicot et al., 2011). Furthermore, the alliance is related to a positive outcome in treatment of BPD (Barnicot et al., 2012). Among PD\SUD patients receiving MBT one study found that clear communication and affect focus on influenced positively completion of therapy (Philips et al., 2017). Likewise, qualitative investigations of patients receiving MBT have underlined the importance of building trust (O Lonargain et al., 2017) and the experience of being understood (Johnson et al., 2016). We find it interesting that patients trust more therapists who address the relationship, while at the same time expressing that it is unnecessary and even uncomfortable to explicitly talk about the relationship with their therapists. According to Bateman (2010–2018), one of the most common errors therapists do is not addressing the relationship. Since the main mechanism of change in MBT is said to be a focus on mentalizing while activating the attachment relationship (Fonagy et al., 2015), it is a paradox that therapists sometimes avoid this topic. Perhaps when patients show discomfort, therapists avoid pushing this further, even though theoretically, mentalizing the therapeutic relationship is of key importance. Our findings suggest that holding focus on the relationship is important. These patients are vulnerable for alliance ruptures and this fate is intensified when SUD patients have the presence of cluster B traits (Olesek et al., 2016). Systematic reviews have shown how important the alliance rupture repair is in therapy (Eubanks et al., 2018). Patients themselves experience clear communication and affect focus as important, our manuals encourage us to manage these phenomena, and the phenomenology of core problems in PD suggests that relational conflicts will be a clinical issue. Our findings support the studies mentioned above and underline the importance of focus on both the patient and the therapist contributions in concert.

### Group Experiences

Our findings suggest that oscillation between bonding and differentiating with co-patients in the group is important for creating positive changes in self-image and for increasing patients’ ability to have multiple perspectives in mind during emotional stress. This resonates well with the idea that the mentalizing is a core component for regulating affect (Philips et al., 2012). When patients are bonding with each other in MBT groups, the danger of them engaging in pseudo-mentalizing processes are at hand, when all feel exactly like the others, therapists will often worry about the lack of differentiating. After all, therapy is not supposed to be a tea party. Our findings suggest that these processes even if they could be understood as pseudo-mentalizing, are vital for the attachment processes in groups and that we probably need to allow them to happen. We suggest that group therapists allow some of these “tea-party” processes to go on, but continue to balance between differentiating and validating processes. These findings also resonate with a qualitative study performed on MBT groups, where they found that keeping authority as a group leader, and keeping at the same time, a not knowing stance is key ingredient for a MBT group therapist (Karterud, 2018). The not knowing stance allows patients to more freely express whatever is on their mind. The risk is that this might favor a more supportive group culture with low collective reflective functioning (Karterud et al., 2019). Moving to an authoritative stance, the therapist would then need to challenge unwarranted assumptions or pretend mode conversation. We notice that patients had no comments on the issue of structure in therapeutic sessions. It is mentioned as a meta-domain in Bateman’s manual (Bateman, 2018) but was not practiced for the individual therapy in this case. It has also been found as significant in a qualitative meta-analysis on client experiences (Levitt et al., 2016). However, the groups were structured (see Karterud, 2015), and patients might have experienced it as “natural.” Structure has been found important in other qualitative investigations on patient experiences in MBT (Dyson and Brown, 2016). Our patients in this study seem to be more preoccupied with affective and relation aspects of the therapy.

### Do Not Leave It to the Patients

Finally, the patients experienced distress when therapists did not address clear “elephants” in the room. This also resonates well with a study on borderline patients who dropped out of group therapy that found a discrepancy between therapist understanding of why patients dropped out and the patients’ own view that too little attention was put on their strong negative emotions (Hummelen et al., 2007). It could be that therapists overestimate these patients when it comes to their capacity to deal with difficult thoughts and feelings. In a review, lack of affective communication was found to be related to dropout from therapy for BPD patients (Barnicot et al., 2011). Some of the PD patients in SUD clinics are often tough on the outside, with distanced attachment strategies, inside though they are struggling with all kinds of negative ruminations about relationships with their therapists. When they have conflicts with group members, they dwell on it long after, and therapists must not overestimate their capacity to deal with these difficult and complicated phenomena by themselves. This also resonates well with a recent study where high-quality and low-quality therapy sessions were analyzed, and it was clear that high-quality MBT involved in confronting and addressing negative and difficult feelings in the room with the patients (Folmo et al., 2019).

### Limitations of the Study

The main limitations of the study are that three out of four authors were closely related to the clinical context and also enthusiastic about MBT. Furthermore, five of 18 patients who were invited to the study did not participate. Although we believe that we could have gotten interesting perspectives from these five, especially on less helpful elements of MBT, these patients had an average time in treatment that was lower than the participants in this study. Thus, our study has probably been successful in collecting subjective experiences from “happy customers” from a successful MBT trial. Main strength of qualitative studies lies in contextualizing clinical trials and process studies (Binder et al., 2016). Qualitative methodology cannot infer on effect or efficacy of a treatment alone and cannot conclude on causal mechanisms in the therapeutic process. Therefore, our findings should be interpreted with caution and can be understood as hypothesis generating. These can be investigated further in studies that utilize methodologies suited to infer causality. Hence, our study is not concluding that all patients receiving MBT prefers transparent, outspoken, and emotionally validating therapist, or that an oscillation between perspective taking and identification in groups is universal helpful elements. We believe that these findings are true for a group of PD\SUD, which could be defined as “happy customers” in a specific clinical context in Bergen, given the set of authors that lie behind this study. However, we are curious on whether process studies on elements in MBT will demonstrate these same mechanisms when utilizing other methods for analyzing and exploring data. We hope our findings can inspire both clinicians and researcher in investigating closer how and why MBT works, and if it works, for PD\SUD patients.

### Implications for Future Research

There is so much more to investigate with MBT for PD\SUD patients. The over-riding question is of course if a RCT might reproduce the favorable outcome of this pilot study. Another urgent question concerns gender. This was a study of female patients and it resonates with the fact that the large majority of patients in borderline treatment trials are females. Actually, one knows very little about treatment course of males with PD/SUD. They are obviously harder to recruit to psychotherapy. Do they also pose other process challenges? This question should have priority for the immediate future.

### In Conclusion

Female patients with SUD/PD that have participated in a well-organized and well-conducted MBT program report that in group therapy the oscillation between collective supportive processes and juggling with different perspectives is of importance. Furthermore, their preferred type of therapists has an interpersonal style of clear communication, tolerance, and validation of affect. They put into words negative unspoken concerns in the group or individual sessions. These therapists mentalize the therapeutic relationship. This is a small qualitative investigation of 13 patients in a SUD clinic in a medium-sized city of Norway, and these findings should be investigated in other clinical contexts and with different methodological approaches.

## Data Availability

The datasets generated for this study are available on request to the corresponding author.

## Ethics Statement

The Regional Committee for Medical and Health Research Ethics (Region West) approved the study. All participants received written and oral information about the purpose of the study and their voluntary participation. They were informed that they could withdraw from the interview at any time.

## Author Contributions

KM has written the manuscript, had the main role in qualitative analyses, and performed interviews. P-EB has contributed in qualitative analyses, writing, and interviewing. NA has contributed in qualitative analyses, writing and interviewing. SK has contributed in qualitative analyses and writing.

### Conflict of Interest Statement

The authors declare that the research was conducted in the absence of any commercial or financial relationships that could be construed as a potential conflict of interest.
